# Fermentation time Determines Anti-inflammatory and Osteoprotective Activity of Green Tea Kombucha in a Rat Model of Experimental Periodontitis

**DOI:** 10.1007/s12602-026-10937-8

**Published:** 2026-02-09

**Authors:** Everton Cavalcante da Silva, Maria Mayara Nascimento Beserra, Marco Gabriel Silva Leitão, Isabelle de Fátima Vieira Camelo Maia, Bianca Elen de Souza Alves, Paola Gyuliane Gonçalves, Deborah Catharine de Assis Leite, Beatriz Gonçalves Neves, Karuza Maria Alves Pereira, Lidiany Karla Azevedo Rodrigues, Delane Viana Gondim

**Affiliations:** 1 Postgraduate Program in Dentistry, Faculty of Pharmacy, Dentistry and Nursing, Federal University of Ceará, Monsenhor Furtado, S/N, Rodolfo Teófilo, 60010-681 Fortaleza, Ceará Brazil; 2 Postgraduate Program in Morphofunctional Sciences, Faculty of Medicine, Federal University of Ceará, Rua Delmiro de Farias, 1331, 60.430-170 Fortaleza, Ceará Brazil; 3 Postgraduate Program in Pharmacology, Faculty of Medicine, Federal University of Ceará. Coronel Nunes de Melo, Coronel Nunes de Melo, 1127, Rodolfo Teófilo, Fortaleza, 60430-275 Ceará Brazil; 4 Specialization Program in Molecular Biology Applied to Bioinformatics, Federal Technological University of Paraná, Av. Sete de Setembro, 3165, Rebouças, 80230-901 Dois Vizinhos, Paraná Brazil; 5 Postgraduate Program in Health Sciences, Faculty of Medicine, Federal University of Ceará, Av. Comandante Maurocélio Rocha Pontes, 100, Derby, Sobral, Ceará 62042-280 Brazil; 6 Department of Morphology, Faculty of Medicine, Federal University of Ceará, Rua Delmiro de Farias, 1331, Rodolfo Teófilo, Fortaleza, 60430-170 Ceará Brazil

**Keywords:** Periodontitis, Probiotics, Kombucha tea, Alveolar bone loss, Inflammation

## Abstract

This study aimed to characterize the microbial composition of green tea–fermented kombucha at different fermentation times and to evaluate its effects on inflammation and alveolar bone loss in rats with periodontitis. Microbial diversity was first assessed by metagenomic sequencing targeting bacterial 16 S rRNA and fungal 18 S rRNA regions. Sixty male rats were divided into six groups: control, periodontitis without treatment, green tea treatment, and kombucha fermented for 4, 8, or 12 days. Kombucha or green tea was administered daily by oral gavage for 39 days. Periodontitis was induced by ligation of the maxillary second molar on day 28. After euthanasia, hemimaxillae, liver, kidney, and blood samples were collected for analysis. Kombucha fermented for 4 days showed the highest abundance of bacteria from the *Acetobacteraceae* family and yeasts from the *Saccharomycetaceae* family. This fermentation time also produced the most pronounced reduction in periodontal inflammation and alveolar bone loss, with lower expression of tumor necrosis factor-alpha (42,9%) and receptor activator of nuclear factor kappa-B (43,6%), as well as higher expression of osteoprotegerin (approximately 55,4%) in periodontal tissues compared to animals with periodontitis without treatment. Notably, kombucha did not induce renal or hepatic toxicity regardless of fermentation time. These findings suggest that kombucha, particularly after 4 days of fermentation, reduces inflammation and alveolar bone loss without systemic toxicity, supporting its potential as an adjunctive therapy for periodontitis.

**Supplementary Information:**

The online version contains supplementary material available at 10.1007/s12602-026-10937-8.

## Introduction

New therapeutic strategies for preventing and management periodontal diseases are being explored to enhance the efficacy of conventional treatments. These approaches aim not only to modulate the host inflammatory response to periodontopathogens but also to stimulate protective and reparative mechanisms [1, 2].

Among these strategies, probiotics have attracted considerable attention. They are generally safe, well-tolerated, and capable of enhancing the intestinal microbiota, inhibiting pathogen adhesion, and modulating immune responses [3]. For instance, *Lactobacillus reuteri* supplementation prevented bone density loss, reduced intestinal dysbiosis, and improved intestinal barrier function in antibiotic administration in mice [4], as well as reduced bone loss in a glucocorticoid-induced osteoporosis model [5].

Kombucha (KB) is a fermented tea beverage rich in probiotics, organic acids, and polyphenols, exhibits antioxidant, anti-inflammatory, and antimicrobial properties. These bioactivities are particular

ly relevant to periodontitis, a chronic inflammatory disease characterized by microbial dysbiosis, oxidative stress, and connective tissue destruction. Despite its growing popularity and demonstrated systemic health benefits, no studies to date have evaluated the impact of green tea-fermented KB on periodontal tissue. This study is the first to investigate the therapeutical potential of KB fermented for different durations in a rat model of periodontitis, providing novel insights into the modulation of oral inflammation and bone loss.

Traditionally, KB is produced by fermenting black or green tea with sucrose (typically 50 g/L) at room temperature for up to 21 days, using a symbiotic culture of bacteria and yeast (SCOBY) [6, 7]. Its microbial composition evolves dynamically throughout fermentation, influencing its bioactive profile. Previous studies have shown that KB exerts systemic benefits, including antioxidant and anti-inflammatory effects and improvements in metabolic disorders such as hypercholesterolemia and diabetes [8, 9]. However, its effects on oral health remain largely unexplored. Therefore, we hypothesized that oral administration of green tea–fermented KB would attenuate periodontal inflammation and alveolar bone loss, and that these protective effects would be modulated by fermentation duration and microbial dynamics.

## Methods

### Preparation of Green Tea-Fermented KB and pH Measurement

KB was prepared under aseptic conditions using sterilized containers and utensils to prevent contamination. First, 250 mL of distilled water were boiled in a glass Erlenmeyer flask for 15 min to sterilize the liquid and extract tea compounds. After 10 min of boiling, 1.6 g of dried green tea leaves (Leão^®^, Arbessa LTDA., São Paulo, SP, Brazil) were added for infusion. At the end of boiling process, 25 g of granulated sugar (União^®^, Camil Alimentos S.A., São Paulo, SP, Brazil) were dissolved in the hot solution to serve as a fermentation substrate.

The tea infusion was allowed to cool to room temperature (approximately 25^o^ C) to preserve microorganism viability. Once cooled, 10 mL of a starter culture containing the SCOBY were added. Fermentation was carried out at 27 °C, protected from direct light, for 4, 8, or 12 days, following the protocol of Gaggìa et al. (2018) [10]. During fermentation, the KB was stored in sterile Falcon tubes covered with a nonwoven fabric (TNT) secured over the openings. This allowed proper gas exchange while preventing external contamination, ensuring aerobic conditions for optimal microbial activity.

Fermentation times of 4, 8, and 12 days were selected based on typical commercial practices. The 4- to 8-day period represents the time when KB is traditionally cons

umed, balancing acidity and palatability. Inclusion of the 12-day time point allowed to investigate whether extended fermentation alters microbial composition or enhances the therapeutic potential of KB, thereby strengthening the translational relevance of this study for dental applications [11].

The pH was determined using a digital pH meter W3B (Bel Engineering^®^, Monza, Italy). The instrument was calibrated at pH 4.0, 7.0, and 10.0 before use.

### DNA Extraction and Data Analysis of 16 S and 18 S Ribosomal RNA (rRNA) Gene Amplicons

Total genomic DNA was extracted from the cellulosic biofilm of green tea-fermented KB samples collected after 4, 8, and 12 days of fermentation. DNA isolation was performed using the DNeasy PowerSoil Pro Kit (Qiagen, Hilden, Germany), following the manufacturer’s protocol.

For bacterial community profiling, the V4 hypervariable region of the 16 S rRNA gene was amplified by polymerase chain reaction (PCR) using the primers 515 F-Y and 806R [12, 13]. For fungal profiling, the V9 region of the 18 S rRNA gene was amplified using primers EUK1391-F and euk-Br [14]. PCR reaction was carried out in a final volume of 30 µL, containing 2 µL of genomic DNA (5 ng/µL), 0.75 µL of each primer (10 µM), 6.0 µL of GoTaq G2 HotStart (5X) Master Mix (Promega, Madison, WI, USA), 3.6 µL of MgCl₂ (25 mM) (Promega), 0.6 µL of dNTPs (10 mM), 0.2 µL of Taq polymerase (5 U/µL), and 16.1 µL of nuclease-free ultrapure water.Amplifications were performed in an Eppendorf Mastercycler Gradient Thermal Cycler (Eppendorf, Hamburg, Germany) under the following conditions: initial denaturation at 95 °C for 3 min; 35 cycles of 98 °C for 30 s, 55 °C for 30 s, and 72 °C for 45 s; and a final extension at 72 °C for 5 min. Amplicons were visualized on a 2% (w/v) agarose gel and purified with Ampure XP beads (Beckman Coulter, Brea, CA, USA).

Indexed libraries were generated in a secondary PCR using 25 µL of 2X KAPA HiFi Ready Start Mix (Roche, Basel, Switzerland), 5 µL of each Nextera XT index primer (Illumina, San Diego, CA, USA), 5 µL of purified amplicon template, and 10 µL of nuclease-free water. Indexed amplicons were purified using Ampure XP beads, quantified with a Qubit^®^ 2.0 fluorometer (Invitrogen, Carlsbad, CA, USA), and normalized according to Illumina guidelines. Equimolar libraries were pooled, diluted, and denatured prior to sequencing. Paired-end sequencing was performed on an Illumina MiSeq platform using the MiSeq Reagent Kit v2 (300 cycles), following the manufacturer’s protocol.

Raw sequence data were processed using the QIIME 2 software package (version 2023.2). Quality control, dereplication, and chimera removal were performed with the DADA2 plugin (q2-dada2). High-quality reads were resolved into amplicon sequence variants (ASVs), which were taxonomically classified against the SILVA reference database (complete 16 S rRNA gene). For 18 S rRNA reads, analogous steps were applied. Sequences corresponding to chloroplasts, mitochondria, archaea, and singletons were removed prior to downstream analyses. Taxonomic composition and relative abundance profiling were performed using MicrobiomeAnalyst 2.0 (https://www.microbiomeanalyst.ca/). For this analysis, only ASVs with a minimum of 4 read counts and present in at least 10% of samples were retained. Alpha diversity (Shannon and Simpson indices) and beta diversity (Bray–Curtis dissimilarity) were calculated for both bacterial (16 S) and fungal (18 S) datasets using QIIME 2 and visualized with MicrobiomeAnalyst 2.0. Rarefaction was applied to normalize sequencing depth across samples prior to diversity analyses.

### Ethical Statement of in Vivo Research

This study was approved by the Ethics Committee on Animal Research at the Federal University of Ceará (protocol number 20190820-0/2020). The research adhered to the ethical principles outlined in the National Institutes of Health Guide for the Care and Use of Laboratory Animals (NIH Publication No. 8023, revised in 1978) and complied with the regulations set forth by the National Council for the Control of Animal Experimentation (CONCEA). Furthermore, the study was conducted and reported in accordance with the ARRIVE guidelines.

### Animals

Sixty male Wistar rats (6–7 weeks old; 200–250 g) were obtained from the Central Animal Facility of the Federal University of Ceará. Only male rats were used in this study to avoid the potential influence of estrous cycle–related hormonal fluctuations on inflammatory and bone responses, thereby ensuring greater group homogeneity and reducing biological variability. The animals were housed under controlled conditions, with free access to food and water, an ambient temperature of 22–24 °C, and a 12-hour light/dark cycle, with four rats per cage. They were randomly assigned to two experimental sets, each consisting of the following groups, using a computer-generated random order: EP (experimental periodontitis), GT (EP + green tea administration), KB4 (EP + green tea-fermented KB for 4 days), KB8 (EP + green tea-fermented KB for 8 days), and KB12 (EP + green tea-fermented KB for 12 days). The contralateral hemimaxilla of the EP group was considered the control group (C, no intervention) and was used solely as a within-animal reference for descriptive purposes; it was not treated as an independent experimental unit. All statistical analyses were performed at the rat level.

The primary outcome variable was alveolar bone loss (ABL). The secondary outcome variables included inflammatory markers, histopathological changes, and systemic toxicity evaluation. Previous studies have reported that ligature placement induces periodontitis in approximately 80% of animals, with ABL and inflammation as the main outcomes [2, 15]. Therefore, six animals were included in each experimental group.

To assess the sensitivity of the current design, a *post hoc* power analysis was performed based on the ANOVA results for ABL (F = 128.6, *p* < 0.0001; six groups, *n* = 6 per group, α = 0.05). The estimated effect size was η² = 0.96 (corresponding to f = 4.6), resulting in a statistical power greater than 0.99. These findings indicate that the present sample size provides sensitivity to detect biologically relevant differences among groups.

### Experimental Protocol

Following a five-day acclimatization period, animals in the GT, KB4, KB8, and KB12 groups received a daily dose (5 mL/kg; oral gavage) of either fermented green tea or KB for 4, 8, or 12 days, respectively. Treatments were administered for 39 consecutive days [2, 9]. Animals were monitored weekly for signs of distress, including evaluation using the Rat Grimace Scale. No animal met the criteria for exclusion during the experimental period. On day 39, all animals were euthanized under deep anesthesia with an overdose of ketamine (240 mg/kg; Dopalen^®^, Agribands, Paulínia, Brazil) and xylazine (30 mg/kg; Rompum^®^, Bayer Saúde Animal, São Paulo, Brazil), administered intrapertoneally.

Experimental periodontitis was induced on day 28 by placing a cotton ligature around the cervical region of the maxillary left second molars (M2), knotted at the buccal surface of the tooth and maintained in place for 11 days. This ligature-induced periodontitis model was chosen because it reproduces key features of human periodontitis, including inflammatory bone resorption. The procedure was performed under anesthesia, induced by an intraperitoneal injection of ketamine (70 mg/kg) and xylazine (6 mg/kg). Animals were positioned supine on a custom surgical table that allowed stable mouth opening to access the maxillary molars. The procedure was carried out by a single trained operator (E.C.S.), who was blinded to the treatment allocations. Animals were monitored during and after the procedure for signs of distress, and no animals were lost due to the anesthetic or surgical procedures.

The right hemimaxilla of the periodontitis group, which did not undergo any treatment or ligature placement, served as the healthy control for intra-animal comparison.

### Morphometric and Radiographic Analysis

The hemimaxillae were dissected, and soft tissues were carefully removed. The specimens were cleared in 2.5% sodium hypochlorite for one minute and then stained with 1% methylene blue for 10 s to highlight the cementoenamel junction. Subsequently, the hemimaxillae were mounted on histological slides and photographed alongside calibration grids measured in millimeters for standardization. Images were exported and analyzed using the open-source software ImageJ^®^ (NIH, Bethesda, MD, USA) to assess ABL in mm². Measurements were performed specifically in the furcation area of the maxillary left second molar (M2), from the cemento-enamel junction to the alveolar bone crest, providing a consistent and reproducible assessment of bone loss.

Radiographic bone density (RBD) was evaluated using radiographs of the same specimens, obtained at a standardized distance of 30 cm from the X-ray source. Radiographic images were processed with ImageJ software (National Institutes of Health, Washington, DC, USA; version 1.51k) to calculate the mean grayscale values at the furcation area of the M2. These values were normalized using a standardized metal matrix for consistency.

All analyses were conducted by a researcher blinded to the experimental groups (D.V.G.).

### Histometric and Histopathological Analysis

After euthanasia, the hemimaxillae, along with liver, kidney, and blood samples, were collected. The specimens were fixed in 10% buffered formalin, and the hemimaxillae were decalcified in 4% ethylenediaminetetraacetic acid (EDTA). Serial sections, 4 μm thick, were prepared in the mesio-distal direction and stained with hematoxylin and eosin (H&E) for microscopic analysis. Histopathological evaluation was conducted by an experienced pathologist blinded to the treatments (K.M.A.P.), using a binocular microscope (Leica DM 2000, Wetzlar, Germany). Collagen fiber organization in the periodontal ligament was also examined under a fluorescence microscope (Leica DM 2500, Wetzlar, Germany).

For histometric evaluation, sections representing the central buccal-lingual portion of the M2 were selected to ensure consistent orientation across specimens. Digital images were captured using a camera attached to a light microscope (Leica DM 2000, Wetzlar, Germany) at 100x magnification. Standardized measurements were performed by an evaluator blinded to the treatment groups (E.C.S.), using the open-source software ImageJ^®^ (NIH, Bethesda, MD, USA). The area of bone loss in the furcation region of M2 was calculated in µm², and the average of the values from two analyzed sections was recorded for each animal [1].

Histopathological assessment of the liver and kidney tissues was conducted using a scoring system ranging from 0 to 3, where 0 = absent, 1 = mild, 2 = moderate, and 3 = severe, to evaluate the presence of edema and vascular changes [15].

### Immunohistochemistry for Tumor Necrosis Factor (TNF-α), Receptor Activator of Nuclear Factor Kappa Beta (RANK), and Osteoprotegerin (OPG)

Periodontal tissue samples were sectioned into 4 μm slices and mounted on poly-L-lysine-coated slides. The streptavidin-biotin-peroxidase method was used to evaluate the immunoexpression of TNF-α, RANK, and OPG in the samples. After deparaffinization and rehydration, antigen retrieval was performed by incubating the slides in citrate buffer (pH 6.0) at 85 °C for 30 min. Endogenous peroxidase activity was blocked by treating the slides with 3% hydrogen peroxide diluted in PBS for 30 min. Following PBS rinsing, the tissue sections were incubated overnight at 4 °C with primary rabbit antibodies against TNF-α (1:200; NeoBiotechnologies, California, USA), RANK (1:200; Invitrogen, Massachusetts, USA), and OPG (1:200; BiossAntibodies, Massachusetts, USA).

The following day, the sections were treated with the EnVision Plus-HRP system (Dako, Santa Clara, USA) as the secondary antibody. Immunostaining was developed using 3,3’-diaminobenzidine (DAB) (Dako, Santa Clara, USA), and counterstaining was performed with Harris hematoxylin. The slides were then dehydrated through a graded ethanol series, cleared in xylene, and mounted with Entellan (Merck KGaA, Darmstadt, Germany) under a coverslip.

A calibrated examiner used an optical microscope (Leica DM 2000, Wetzlar, Germany) to identify five “hot spot” fields per sample at ×400 magnification. Quantitative analysis of DAB-stained products from immunostaining was performed on digital images captured from at least ten distinct areas per section (four specimens per group) exhibiting the highest immunostaining intensity. Immunohistochemical analyses for TNF-α, RANK, and OPG were conducted at 400× magnification. DAB-stained sections were analyzed in open-source ImageJ (NIH, Bethesda, MD, USA) using a uniform pixel intensity threshold, set from representative sections, to ensure consistent quantification [16]. All analyses were performed by a researcher blinded to the group assignments (D.V.G.).

### Analysis of Serum Hepatotoxicity Markers

Blood samples were centrifuged (1800G; 10 min), and the supernatants were collected and stored at -80 °C for biochemical analysis of aspartate aminotransferase (AST) and alanine aminotransferase (ALT). Serum levels of hepatotoxicity markers were determined according to the manufacturer’s instructions (Labtest, Lagoa Santa, MG, Brazil).

### Statistical Analysis

All results are expressed as mean ± SEM for parametric data or as median (minimum–maximum) for non-normally distributed data. Data normality was assessed using the Shapiro-Wilk test. Parametric data were analyzed using analysis of variance (ANOVA) followed by Tukey’s post hoc test. Non-parametric data were analyzed using the Kruskal-Wallis test, followed by Dunn’s post hoc test. Statistical analyses were conducted using GraphPad Prism 10, with significance set at *p <* 0.05.

## Results

### The Permentation Time of KB Affects the Microbial Diversity.

**Fig. 1:**
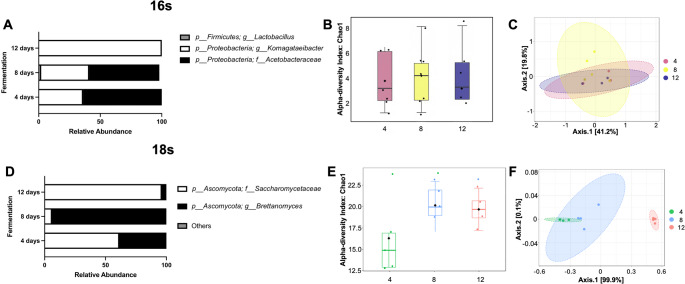
Relative abundance of bacterial (**a**) and yeast (**d**) communities in green tea-fermented kombucha at 4, 8, and 12 days, presented at both the phylum and genus levels. Alpha-diversity of bacterial (**b**) and fungal (**e**) communities. Beta-diversity analysis of bacterial (**c**) and fungal (**f**) communities. 4 days= green tea-fermented kombucha for 4 days; 8 days= green tea-fermented kombucha for 8 days; 12 days= green tea-fermented kombucha for 12 days

A heterogeneous community of bacteria and yeasts was detected in samples fermented for 4 (pH 3.4), 8 (pH 2.6), and 12 (pH 2.1) days. Among bacterial taxa, the phylum *Proteobacteria* was predominant at days 4 and 8, with members of the family *Acetobacteraceae* representing approximately 70% and 60% of the community, respectively, while *Komagataeibacter* accounted for ~ 30% and ~ 40% at these time points. The phylum *Firmicutes*, represented by the genus *Lactobacillus*, appeared only after 8 days of fermentation, though at low relative abundance. By day 12, *Komagataeibacter* dominated, constituting 100% of the bacterial community (Fig. 1A).

Alpha diversity of the 16 S V4 region microbiome (Fig. 1B) varied with fermentation time. The highest richness and diversity were observed on day 8, followed by day 4. The decline in richness at day 12 may be related to medium acidification or substrate competition, creating unfavorable conditions for the growth of some bacterial taxa. Beta diversity analysis, using PCoA and PERMANOVA, revealed distinct microbial profiles associated with fermentation time (Fig. 1C). Samples fermented for 4 days showed the greatest similarity to each other, whereas those fermented for 8 days displayed a distinct microbial composition. Samples fermented for 4 and 8 days were more similar to each other than to those fermented for 12 days (p-values: 4 vs. 8 = 0.003; 4 vs. 12 = 0.02; 8 vs. 12 = 0.03).

The relative abundance of fungal taxa also shifted throughout fermentation. *Saccharomycetales* predominated on days 4 and 8, whereas *Pichiaceae* became more abundant on day 12, indicating that fermentation time directly shapes fungal community composition.

Within fungal communities, the phylum *Ascomycota*, particularly the family *Saccharomycetaceae*, showed higher relative abundance at days 4 and 12 (~ 65% and ~ 95%, respectively). On day 8, the genus *Brettanomyces* dominated, reaching ~ 95% relative abundance (Fig. 1D), indicating a temporal shift in yeast composition during fermentation.

Overall, both richness and variability peaked on day 8. Clustering analysis further showed that samples from days 4 and 8 grouped more closely, whereas day 12 exhibited a distinct fungal community structure (p-values: 4 vs. 8 = 0.91; 4 vs. 12 = 0.02; 8 vs. 12 = 0.008).

### Green-tea KB Fermentation Time Influences Inflammation and Bone Loss in Rats with Periodontitis

**Fig. 2:**
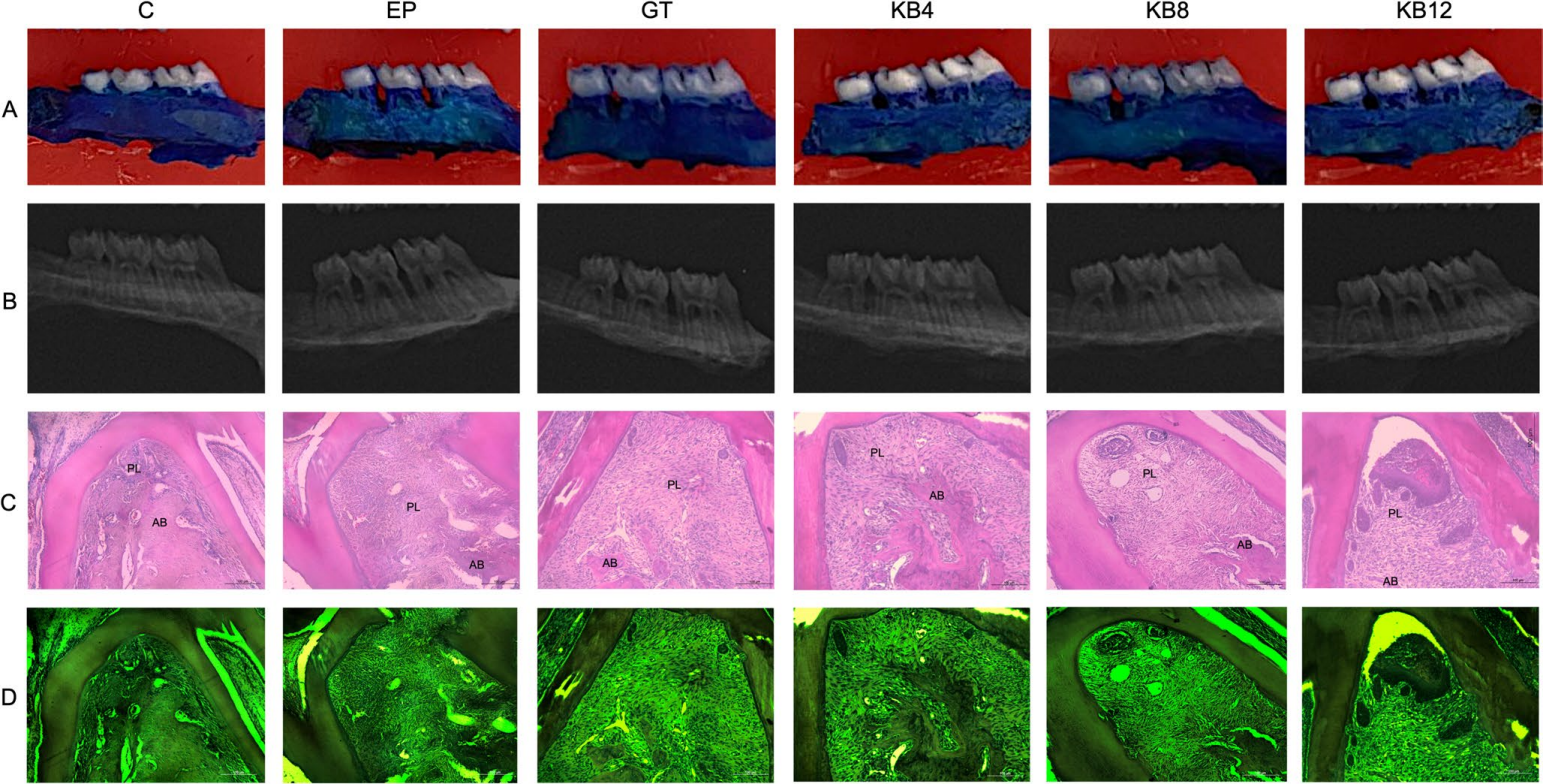
Macroscopic (**a**) and radiographic (**b**) aspects of hemimaxillae in animals with periodontitis that received green tea or kombucha at different fermentation times. Photomicrographs (**c**) and autofluorescence of periodontal ligament fibers in the furcation area of the maxillary second molar in animals with periodontitis showing collagen disorganization (**d**) in the furcation area in maxillary second animals with periodontitis that received green tea or kombucha at 4, 8, or 12 days of fermentation (Hematoxylin and eosin stain; 200x magnification). Data are presented as mean ± SEM. One-way ANOVA, Tukey’s test. Symbols indicate statistical significance: *p < 0.05 *versus* C; ^#^ *p* < 0.05 *versus* EP; ^$^ *p* < 0.05 *versus* GT; ^&^ *p* < 0.05 *versus* KB4; ^+^ *p* < 0.05 *versus* KB8. AB= alveolar bone; PL= periodontal ligament

**Fig. 3:**
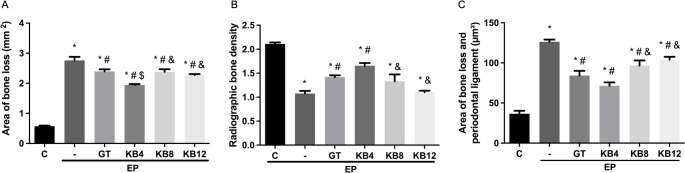
Macroscopic (**a**) and radiographic bone density (**b**) analysis of area of bone loss. (**c**) Histometric analysis of area of bone loss and periodontal ligament. All analyses were performed in animals with periodontitis that received green tea or kombucha at 4, 8, or 12 days of fermentation. Results are presented as mean ± SEM. * *p <* 0.05 *versus* C. ^#^ *p <* 0.05 *versus* EP. ^$^ *p <* 0.05 *versus* GT. ^&^ *p <* 0.05 *versu*s KB4. C= Control; EP= experimental periodontitis; GT= green tea; KB4 = green tea-fermented kombucha for 4 days; KB8 = green tea-fermented kombucha for 8 days; KB12 = green tea-fermented kombucha for 12 days (One-way ANOVA; Tukey test)

A significant increase in ABL was observed in all experimental groups compared to the C group (0.570 ± 0.023 mm²; *p <* 0.001). However, animals with periodontitis that received green tea or KB showed a reduction in ABL compared to the EP group (*p* = 0.012 and *p* < 0.001, respectively). Notably, the KB4 group (1.946 ± 0.028 mm²) exhibited a significant reduction in ABL compared to the GT (2.396 ± 0.067 mm²; *p =* 0.001), KB8 (2.381 ± 0.086 mm²; *p =* 0.001), and KB12 (2.275 ± 0.031 mm²; *p =* 0.02) groups (Figs. 2 and 3A).

Regarding grayscale variation in the furcation region of the upper left M2, groups that received green tea or KB fermented for 4 days (1.656 ± 0.055) showed a significant increase in RBD compared to the EP group (1.076 ± 0.053; *p* < 0.001). Animals with periodontitis that received KB fermented for 4 days exhibited a significant increase in the same parameter compared to the KB8 (1.330 ± 0.143; *p =* 0.04) and KB12 (1.102 ± 0.033; *p* < 0.001) groups. No statistically significant differences were found between the KB4 and GT groups or between the KB8 and KB12 groups (Figs. 2 and 3B).

In the furcation area, a significant increase in bone and ligament loss was observed in all animals subjected to experimental periodontitis compared to the control group (36.58 ± 3.769 μm²; *p <* 0.001). However, treatment groups exhibited less bone and ligament loss, with the KB4 group (71.43 ± 4.236 μm²) demonstrating significantly less loss compared to the KB8 (96.71 ± 6.318 μm²; *p =* 0.004) and KB12 (104.0 ± 3.568 μm²; *p <* 0.001) groups (Figs. 2 and 3C).

The role of TNF-α in promoting osteoclastogenesis through the RANK/RANKL pathway was evident in our study, as TNF-α expression was significantly reduced in the KB4 group compared to the EP group (9.443 ± 0.222; *p <* 0.001). The reduction in TNF-α expression in the KB4 group correlates with the observed reduction in ABL, suggesting that early-stage fermentation of KB may inhibit the inflammatory cascade contributing to bone resorption.

**Fig. 4:**
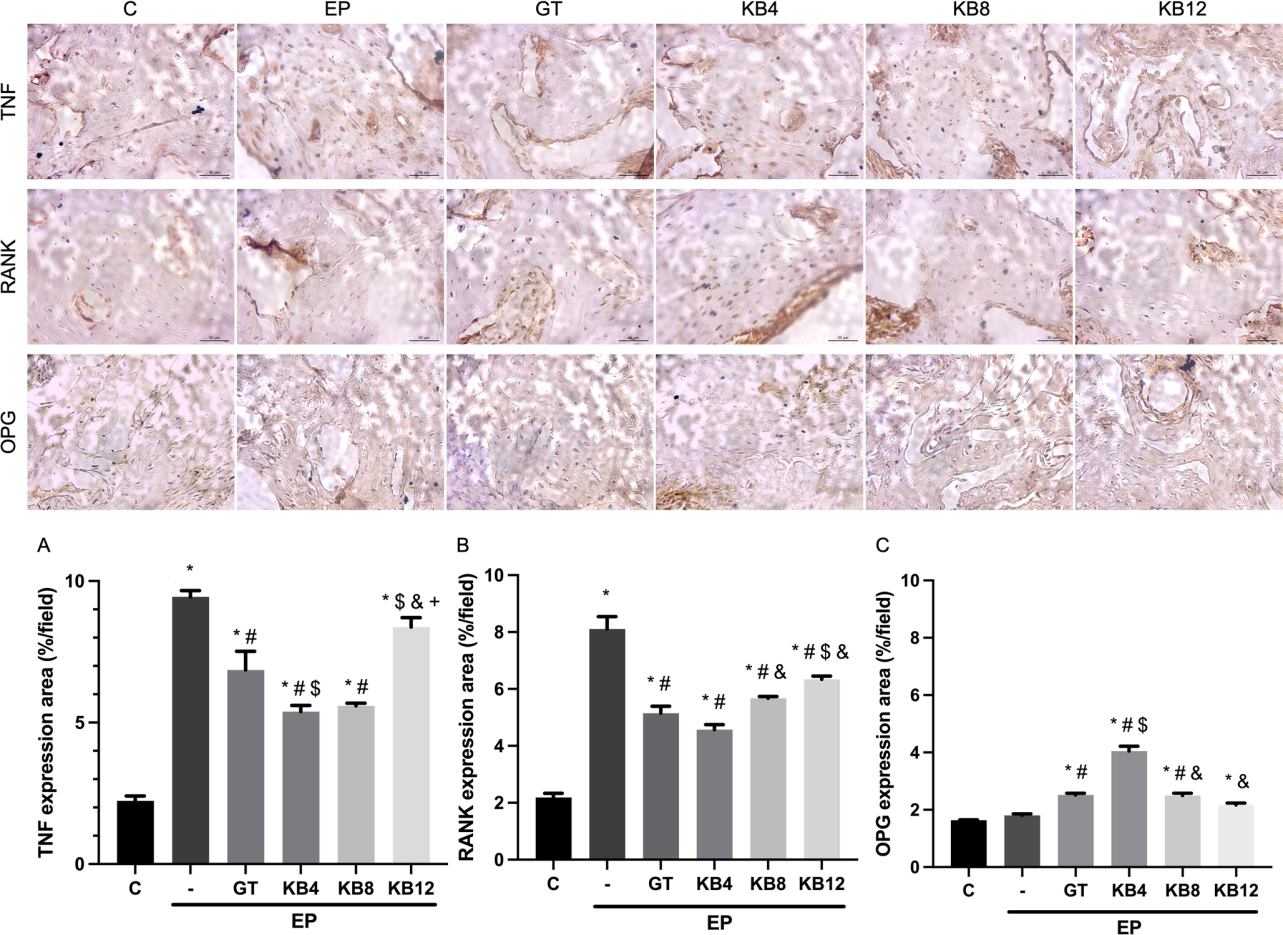
Photomicrographs and immunohistochemical analyses for TNF-α (**a**), RANK (**b**), and OPG (**c**) in the periodontium. Results are presented as mean ± SEM. * *p <* 0.05 *versus* C. ^#^ *p <* 0.05 *versus* EP. ^$^ *p <* 0.05 *versus* GT. ^&^ *p* < 0.05 *versus* KB4. ^+^ *p <* 0.05 *versus* KB8. C= Control; EP= experimental periodontitis; GT= green tea; KB4 = kombucha fermented from green tea for 4 days; KB8 = kombucha fermented from green tea for 8 days; KB12 = kombucha fermented from green tea for 12 days (one-way ANOVA; Tukey’s post hoc test)

Moreover, OPG expression, which acts as a decoy receptor for RANKL and inhibits osteoclastogenesis, was significantly increased in the KB4 group (4.044 ± 0.163) compared to the GT (2.515 ± 0.064, *p* < 0.001), KB 8 (2.45 ± 0.079, *p* < 0.001), and KB12 groups (2.500 ± 0.079, *p* < 0.001) (Fig. 4C).

Although KB8 and KB12 showed statistically significant differences compared to other groups, their biological relevance appears limited when contrasted with KB4. The KB4 group not only achieved statistical significance but also displayed a more pronounced protective effect on bone and periodontal tissues, whereas the changes observed in KB8 and KB12 likely reflect less favorable microbial and metabolic conditions.

### Green Tea-Fermented KB Did Not Cause Systemic Toxicity

In addition to its therapeutic effects on periodontitis, we evaluated the systemic safety of green tea-fermented KB. Histopathological analyses of kidney tissues revealed no significant differences between the EP group and the groups treated with green tea or KB, indicating that KB consumption did not result in systemic toxicity (Table 1). However, the GT group exhibited higher edema scores compared to the KB4 and KB8 groups, and the presence of hemorrhage in the hepatic sinusoids was higher in the GT group compared to the KB4 group.

**Table 1:** Semiquantitative histopathological analysis of the kidney and liver in animals with periodontitis treated with green tea or Kombucha fermented for 4, 8, or 12 days

Groups	Edema			Vascular changes	
	Kidney	Liver		Kidney	Liver
EP	1 (0–1)	1 (0–1)		0 (0–0)	1 (0–1)
GT	1 (0–1)	2 (1–2)		0 (0–1)	1 (0–1)
KB4	0 (0–1)	0 (0–0)^$^		0 (0–0)	1 (1–1)^$^
KB8	0 (0–1)	0 (0–1)^$^		0 (0–1)	1 (1–1)
KB12	0 (0–0)	1 (0–1)		0 (0–1)	1 (0–1)

Each group contained six animals. Association analyses were conducted using the Kruskal-Wallis test. When significant, Dunn’s post hoc test was applied for multiple group comparisons. Results are presented as median (minimum- maximum). *EP* experimental periodontitis, *GT* green tea, *KB4* kombucha fermented from green tea for 4 days, *KB8 *kombucha fermented from green tea for 8 days, *KB12* kombucha fermented from green tea for 12 days

^$^ Statistically significant differences vs. GT

**Fig. 5:**
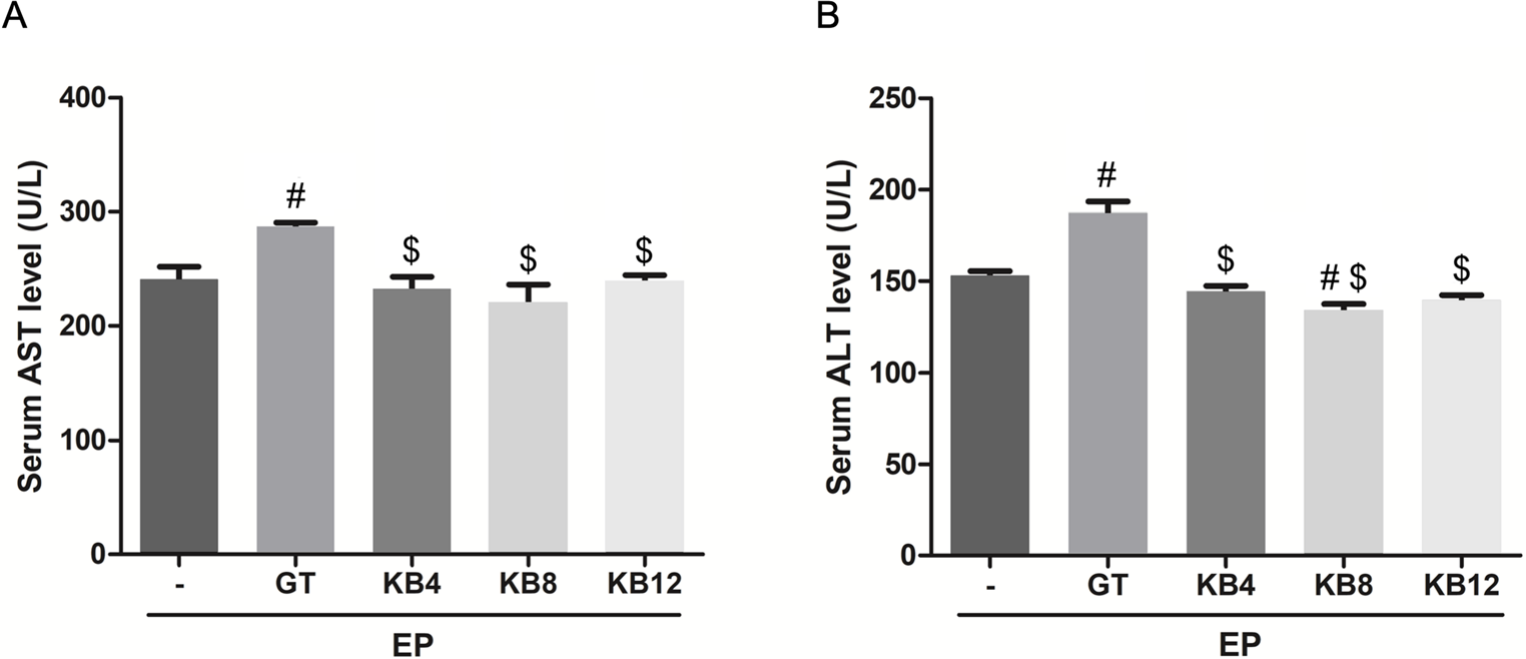
Serum AST (**a**) and ALT (**b**) levels in animals with periodontitis treated with green tea or kombucha fermented for 4, 8, or 12 days. Results are presented as mean ± SEM. ^#^ *p <* 0.05 vs. EP; ^$^ *p* < 0.05 vs. GT. EP = experimental periodontitis; GT = green tea; KB4 = kombucha fermented from green tea for 4 days; KB8 = kombucha fermented from green tea for 8 days; KB12 = kombucha fermented from green tea for 12 days (one-way ANOVA; Tukey’s post hoc test)

A significant increase in ALT levels was observed in the GT group compared to the EP group (*p* < 0.0001). Animals receiving daily green tea administration also exhibited higher ALT levels compared to those receiving KB at various fermentation times (*p <* 0.0001). Additionally, daily administration of KB, after eight days of fermentation, significantly reduced ALT values compared to the EP group (*p* = 0.01). Lastly, consuming only green tea resulted in a significant increase in AST levels compared to the other groups. However, no difference was observed between animals that received KB and those in the EP group (Fig. 5A and B).

The presence of hepatic tissue edema was significantly higher in the GT group compared to the KB4 and KB8 groups. At the same time, another evaluated hepatic parameter, the presence of hemorrhage in the sinusoids, was also statistically higher in animals treated with green tea when compared to those receiving daily administration of KB fermented for 4 days.

## Discussion

To the best of our knowledge, this is the first study to evaluate the effects of green tea-fermented KB at different fermentation times (4, 8, and 12 days) on experimental periodontitis in rats. Our findings demonstrate that KB consumption, particularly after 4 days of fermentation, exerts significant anti-inflammatory and anti-resorptive effects, as evidenced by reduced ABL and improved RBD, without inducing hepatic or renal tissue damage. These findings are in line with other probiotic-based interventions, such as *Lactobacillus reuteri* supplementation and milk kefir administration, which have also demonstrated the ability to reduce bone loss and periodontal inflammation in experimental models [2, 4].

Changes in microbial diversity during fermentation were accompanied by significant differences in ABL and inflammatory responses in rats with periodontitis. The anti-resorptive effect of KB4 is likely mediated by modulation of the RANK/RANKL/OPG signaling pathway, as reflected by increased OPG expression and reduced levels of TNF-α and RANK. This suggests that KB fermented for shorter periods may attenuate osteoclastogenesis and bone resorption in inflammatory conditions such as periodontitis.

The more pronounced effects observed with KB fermented for 4 days may be explained by the distinct microbial and biochemical profile characteristic of early fermentation. The comparatively lower diversity at day 4 may be explained by the higher sugar concentration and shorter fermentation time, which limit microbial overgrowth. At this stage, a balanced community of *Acetobacteraceae* and *Saccharomycetaceae* predominates, capable of producing bioactive compounds such as bacterial cellulose and levan with known anti-inflammatory properties. Concurrently, shorter fermentation prevents excessive acidification and loss of microbial diversity, maintaining favorable conditions for metabolite production.

Furthermore, green tea polyphenols are more abundant and less degraded during early fermentation, whereas prolonged fermentation promotes their progressive microbial transformation into smaller phenolic acids, which may alter bioactivity. These microbial transformations can also affect the bioavailability of catechins, since microbe-derived metabolites may display distinct anti-inflammatory and antioxidant properties compared to native polyphenols. Together, these mechanisms likely contribute to the stronger anti-inflammatory and anti-resorptive effects observed in the KB4 group. Additionally, KB4 and KB8 exhibited hepatoprotective effects, with significantly less hepatic edema and sinusoidal hemorrhage compared to the GT group, highlighting a more favorable safety profile.

Our data are consistent with evidence showing that modulation of inflammatory mediators, particularly TNF-α, influences bone metabolism by enhancing RANKL signaling and osteoclastogenesis [17, 18]. Indeed, TNF-α not only promotes RANK expression in osteoclast precursors but also enhances RANKL production, driving osteoclast differentiation and activity [19, 20]. This aligns with our findings, where TNF-α downregulation and OPG upregulation in KB-treated groups correlated with a significant reduction in ABL.

The microbial analysis revealed that KB harbored microorganisms predominantly from the phyla *Proteobacteria*,* Firmicutes*,* and Ascomycota*, with their relative abundance modulated by fermentation time. This aligns with previous studies indicating that the KB microbial community is shaped by both the tea substrate and fermentation conditions [21, 22].

The predominance of *Acetobacteraceae*, particularly *Komagataeibacter*, is notable given their role in synthesizing bioactive compounds such as bacterial cellulose and levan. Levan has been shown to possess antioxidant and anti-inflammatory properties by modulating immune responses and reducing pro-inflammatory cytokines [23]. Similarly, bacterial cellulose produced by *Komagataeibacter* contributes to wound healing and immunoregulation [24, 25].

The presence of fungi from the phylum *Ascomycota* further supports KB’s functional properties, given its role in producing emodin, an anthraquinone with anti-inflammatory, antibacterial, and antitumor activities. Emodin mediates its effects through inhibition of Factor nuclear kappa B and NLRP3 pathways and modulation of PI3K/AKT and JAK/STAT3 signaling [26–28].

While green tea has documented anti-inflammatory and antioxidant properties, excessive intake of its polyphenols, especially catechins, has been associated with hepatotoxicity [29, 30]. In this study, the GT group exhibited higher levels of AST and ALT, along with more pronounced hepatic histopathological alterations, compared to KB groups. AST and ALT are widely used biomarkers of liver function, and elevated serum levels indicate hepatocellular damage. These results suggest that the fermentation process may attenuate the potential hepatotoxic effects of green tea, likely through microbial biotransformation of polyphenols into less toxic and more bioavailable compounds [8, 31].

Interestingly, while longer fermentation (12 days) is associated with increased antioxidant capacity due to the release of low-molecular-weight phenolic compounds [31]. However, a study that explored the effects of fermentation time related that the first seven days of fermentation demonstrate antioxidant properties [32]. Our data indicate that the most pronounced anti-inflammatory and anti-resorptive effects occurred with KB fermented for 4 days, whereas the changes observed in KB8 and KB12 likely reflect less favorable microbial and metabolic conditions. Although KB8 and KB12 showed significant differences compared to other groups, their biological relevance appears limited when contrasted with KB4. This suggests that an increased fermentation time could potentially diminish certain beneficial properties, depending on the therapeutic target.

It is also important to note that the present study was conducted exclusively in male rats. Considering that sex hormones, particularly estrogen, have a well-established influence on bone metabolism and immune response [33, 34], future studies should investigate whether KB exerts similar protective effects in females, particularly in postmenopausal models or in conditions with estrogen deficiency.

In summary, green tea-fermented KB, especially when fermented for 4 days, reduced periodontal bone loss by modulating inflammatory and bone remodeling pathway. It demonstrated anti-inflammatory, anti-resorptive, and hepatoprotective effects without causing systemic toxicity. These findings support its potential as a safe functional food for managing periodontitis.

### Limitations and Future Directions

This study has some limitations. It was conducted only in male rats, preventing the evaluation of sex-related differences in the response to KB. The chemical characterization of fermentation-derived metabolites was not performed, which limits the understanding of the link between fermentation time and therapeutic effects. The identification of the cementoenamel junction may involve some subjectivity, although this was minimized by blinded assessments and complementary analyses. Finally, while the animal model provided mechanistic insights, clinical studies are still required to confirm the translational potential of KB. Future research should include both sexes, apply metabolomic approaches to identify bioactive compounds, and evaluate the clinical efficacy and safety of KB as an adjunctive strategy for periodontal therapy.

## Supplementary Information

Below is the link to the electronic supplementary material.

Supplementary Material 1

## Data Availability

Data will be made available on request.

## Declarations

All experimental procedures adhered to the ethical principles outlined in the National Institutes of Health Guide for the Care and Use of Laboratory Animals (NIH Publication No. 8023, revised 1978), complied with the guidelines of the National Council for the Control of Animal Experimentation (CONCEA), and were approved by the Ethics Committee on Animal Research at the Federal University of Ceará (protocol number 20190820-0/2020).
