# Supplementary figures and images for: Fermentation time Determines Anti-inflammatory and Osteoprotective Activity of Green Tea Kombucha in a Rat Model of Experimental Periodontitis
Source: Probiotics Antimicrob Proteins. 2026 Feb 9;18(5):7089–100. doi: 10.1007/s12602-026-10937-8 (PMC13368972; doi:10.1007/s12602-026-10937-8)

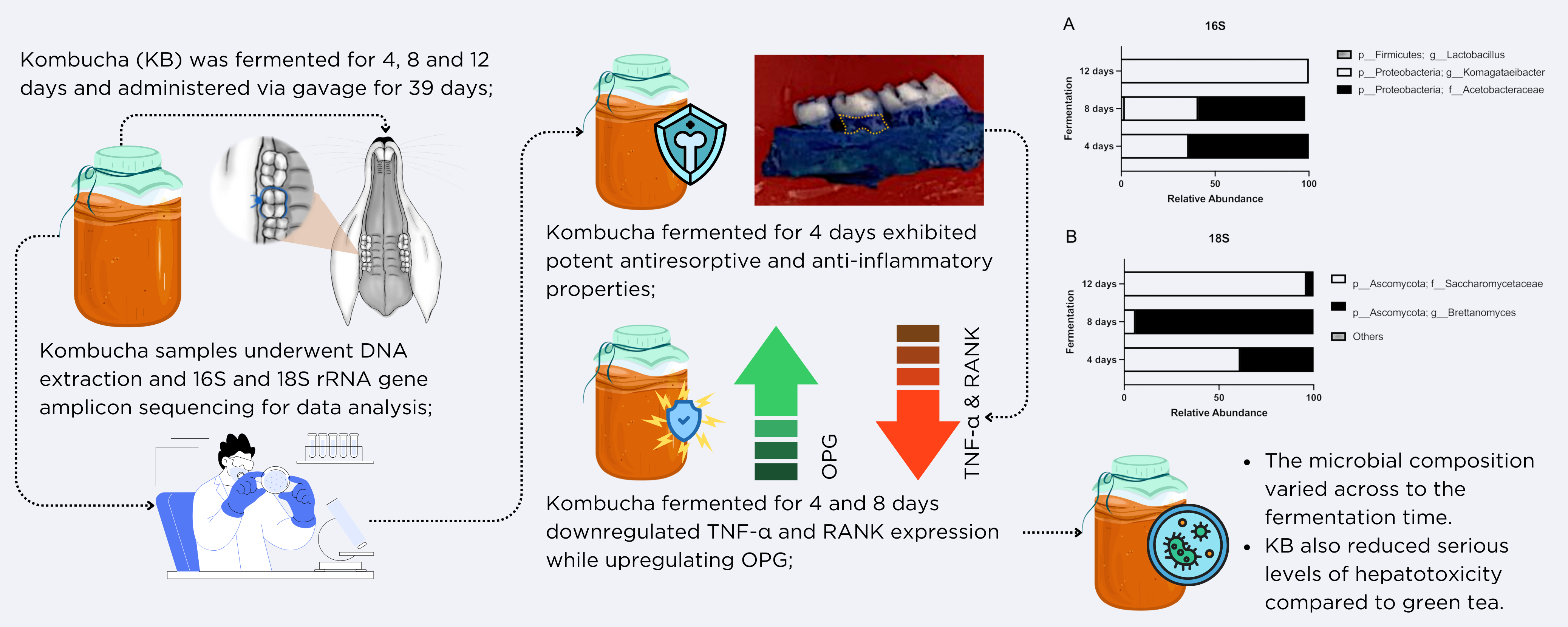

Supplement: Supplementary file 1 — Supplementary Material 1 [file 12602_2026_10937_MOESM1_ESM.tif]
